# Drug-Specific Global Attentional Bias in Females with Drug Use Disorder: Response Slowing Under Short but Not Long Cue Exposure

**DOI:** 10.3390/brainsci15101127

**Published:** 2025-10-21

**Authors:** Biye Wang, Tao Tao, Jian Liu, Zequn Wang, Qing Ren, Wei Guo

**Affiliations:** 1 College of Physical Education, Yangzhou University, Yangzhou 225127, China; wangbiye@yzu.edu.cn (B.W.); mx120240506@stu.yzu.edu.cn (T.T.); qingrenyzu@163.com (Q.R.); 2 Nanjing Compulsory Isolation Drug Rehabilitation Center, Nanjing 211806, China; xichuliusha@163.com (J.L.); 13373680018@163.com (Z.W.)

**Keywords:** attentional bias, drug dependence, dot-probe task

## Abstract

Background/Objectives: Attentional bias toward drug-related cues is a characteristic of drug dependence and plays a detrimental role during drug withdrawal. The present study examined attentional bias in female individuals with drug dependence. We focused on its temporal and spatial characteristics using drug-related and negative emotion dot-probe tasks. Methods: Fifty-one female participants with drug dependence (mean age = 24.71 ± 7.58 years) took part in the study. These participants were primarily dependent on methamphetamine and novel psychoactive substances. They completed tasks with two cue exposure durations (500 ms and 2000 ms) under three spatial conditions: match, mismatch, and neutral. Results: Results indicated that a global attentional bias toward drug-related cues, rather than a location-specific bias, was evident during the short cue exposure (500 ms), regardless of spatial alignment (*ps* < 0.05), whereas no bias was observed during the sustained attention stage (2000 ms). No attentional bias was observed for negative emotional stimuli, highlighting the stimulus-specific nature of this effect. Conclusions: These findings further support the incentive sensitization model of addiction, showing that interference from drug-related items, regardless of the specific orientation of attention, primarily drives short cue exposure attentional bias in females.

## 1. Introduction

Attentional bias toward drug-related stimuli has been proposed as a characteristic of addictive behavior [1], reflecting the preferential allocation of attention to environmental stimuli associated with an individual’s goals or motivational states [2]. For individuals with drug dependence, these stimuli are primarily drug-related cues. Individuals with substance use disorders may also exhibit attentional bias toward negative emotional cues [3], as negative emotions elicited by drug withdrawal can further promote drug-seeking and use, consistent with negative reinforcement frameworks [4]. This process is thought to be maintained by a reciprocal feedback loop between craving and attentional bias. Heightened craving enhances attentional capture by drug cues [5,6], which in turn increases craving and perpetuates drug-seeking behavior [7]. However, it is important to note that some studies have found the relationship between attentional bias and craving to be limited or inconsistent [7,8,9]. Regardless, investigating attentional bias provides an objective measure for assessing the psychological rehabilitation status of individuals with drug dependence.

Numerous studies have reported two types of attentional bias effects: the global effect and the location-specific effect [10,11,12,13,14,15]. Della Libera et al. found a global attentional bias toward drug-related cues [15]. This phenomenon is characterized by longer response times for cue pairs containing drug-related images compared to neutral image cue pairs, regardless of whether the drug cue’s location aligns with the subsequent target location. In other words, individuals with drug addiction show slower response times irrespective of the spatial alignment between the drug-related cue and the target. This can be explained by the higher level of interference that drug-related cues cause in individuals with addiction, possibly due to the activation of drug-related thoughts and concerns that require suppression, along with a greater allocation of attentional resources [16]. The location-specific effect of attentional bias is often revealed through dot-probe tasks, where drug users’ attention is rapidly captured by drug-related cues, remains focused on these cues for longer durations, and facilitates faster responses to subsequent probes appearing at the same location [17]. Conversely, when targets appear in a location different from the drug-related cue, drug users show difficulty disengaging attention, as reflected by slower reaction times [18]. Converging evidence from event-related potentials (ERPs) shows that drug-related pictures elicit shorter P1 latencies and larger N1, N2, and P2 amplitudes, suggesting short cue exposure attentional capture and greater consumption of attentional resources [19]. Attentional bias in this study is operationalized as relative slowing of reaction times. The incentive sensitization model of addiction explains these phenomena. It proposes that repeated drug use makes the brain’s reward system hypersensitive to the motivational effects of drugs and associated cues, thereby generating attentional bias and promoting continued drug use [20].

Studies using stimulus durations of 200 ms, 500 ms, and 1500 ms show attentional bias in individuals with drug dependence. The bias appears mainly during the initial orienting stage rather than during sustained attention [21]. These findings can be interpreted through load theory. Longer cue durations may induce higher perceptual load. As a result, available perceptual capacity is largely consumed by relevant stimuli. Irrelevant distractors are filtered out during short cue exposure of processing, reducing their impact on subsequent task performance [22,23]. Collectively, these results suggest that the duration of drug-cue exposure may influence both the detection and direction of attentional bias.

However, research on the effects of cue exposure duration has shown inconsistent findings [2,21,24]. For instance, Branchadell et al. used a dot-probe task with cue durations of 500 ms and 2000 ms. The 500 ms duration was taken to reflect the initial stage of attention, and the 2000 ms duration to reflect sustained attention. They found that participants responded significantly faster to probes replacing drug-related pictures than to probes replacing matched control pictures in the 500 ms condition. This effect was not observed in the 2000 ms condition [24]. Although this pattern does not fully align with some earlier findings [21], it suggests that attentional bias is more likely to emerge under short cue conditions. Importantly, these studies measured attentional bias only by comparing neutral trials with trials where drug cues and probes were spatially congruent, without considering incongruent trials. By contrast, another study using 500 ms cue exposure found that drug-related cues slowed reaction times regardless of their spatial congruence with the probe [25], and He et al. reported slower reaction times in incongruent trials compared with neutral trials, whereas no significant difference was observed between congruent and neutral trials [18].

Research on attentional bias toward negative emotional cues has also shown mixed results. Following the administration of 20 mg of methamphetamine, participants were more likely to direct their initial gaze toward negative facial expressions, indicating negative emotional attentional bias [26]. Similarly, abstinent heroin users exhibited heightened attention to negative emotional stimuli [27]. However, other behavioral and electrophysiological studies have failed to support the existence of such a bias. Although difficulty disengaging attention from drug-related cues has been observed, no significant differences were found between negative and neutral facial expressions, regardless of whether cues and targets were spatially congruent or incongruent [18]. Moreover, abstinent heroin users and healthy controls did not differ in early posterior negativity (EPN) amplitudes for emotional versus neutral stimuli. In contrast, heroin users showed stronger EPN modulation to heroin-related cues, a pattern absent in the control group [28]. These findings suggest that attentional bias in individuals with drug dependence may be specific to drug-related cues. It does not appear to extend to negative emotional stimuli. At the same time, a 500 ms cue presentation may be insufficient to fully capture attentional processing of negative emotional cues, and this possibility cannot be ruled out [29].

Another limitation of prior research is the overrepresentation of male participants, with relatively few studies focusing on attentional bias in female individuals. Specifically, in the study by Branchadell et al., of the 47 participants, only 9 were female [24]. In a study on opioid-dependent individuals, among the 19 participants, only 4 were female [21]. He et al.’s research, on the other hand, focused solely on male participants [18]. However, evidence suggests that females differ from males in drug use behaviors [30]. These differences may also be influenced by sex differences in stress-related systems, which can contribute to greater vulnerability to addiction in females, although this varies depending on the context [31]. For instance, females may be more likely to report using substances to cope with emotional problems, trauma, or stress [32], though men may also use substances for similar reasons, particularly in response to specific stressors. A study focused on females with methamphetamine addiction found that this group, similar to males, exhibited drug-related attentional bias. Notably, this study used a cue exposure duration of 1000 ms and calculated attentional bias based on the difference between congruent and incongruent conditions, which differs from the methodology used in studies predominantly involving male participants. As such, direct comparisons with other studies are challenging [14]. Additionally, research in other fields, such as anxiety, has shown that females and males differ in the time course of attentional bias and visual processing [33]. Therefore, extending research to this population is important, as it helps address the imbalance in the literature and offers a more comprehensive understanding of attentional bias in individuals with drug use disorders.

In summary, to address inconsistencies in previous research, the present study analyzed attentional bias under different cue exposure durations. This study also considered the separability of global and location-specific attentional biases. The cues included drug-related pairs (in the drug task), negative emotional faces pairs (in the emotion task), and neutral pairs (in both tasks). Further distinctions were made between trials where the cues and targets were spatially matched or mismatched. Additionally, the study aimed to partially address the scarcity of research focusing on female individuals with drug dependence. Using both drug- and emotion-related dot-probe tasks, two cueing conditions (500 ms and 2000 ms) were employed to compare reaction times across three spatial conditions: cues matched with the target, cues mismatched with the target, and neutral trials. We hypothesized that individuals with drug dependence would exhibit both global and location-specific attentional biases toward drug-related cues. These biases would be reflected in preferential processing of drug-related stimuli during the short cue exposure condition (500 ms), resulting in increased perceptual load and slower reaction times. However, under the higher perceptual load of the 2000 ms cue condition, we expected that reaction times would remain unaffected, as participants would have more time to allocate their attentional resources.

## 2. Materials and Methods

### 2.1. Participants

The a priori sample size was calculated using G*Power software (version 3.1). The effect size was set at *f* = 0.25, with *α* = 0.05 and power = 0.95. The required minimum sample size was 44 participants. Therefore, 52 females with drug dependence were recruited from a rehabilitation center in Jiangsu, where they were subject to a controlled environment, with regulated daily routines and lifestyles, as part of a compulsory detoxification program. One participant withdrew from the experiment due to other task arrangements, leaving 51 participants included in the final analysis (mean age = 24.71 ± 7.58 years; mean duration of drug use = 5.51 ± 4.82 years). Of the participants, 6 had methamphetamine dependence, 17 had dependence on etomidate, 5 were dependent on novel psychoactive substances, and 23 had multiple drug dependencies. The mean duration of drug use was 5.44 ± 4.84 years, and the mean duration of abstinence was 6.20 ± 5.97 months. The 13-item Obsessive Compulsive Drug Use Scale (OCDUS) was used to assess participants’ self-reported drug craving. Demographic information is presented in Table 1. All participants were fully informed, both in writing and orally, about the voluntary nature and confidentiality of their participation. They were assured that refusing or withdrawing from the study would not result in any penalties, and an anonymous identification number was used to alleviate any concerns. All participants provided written informed consent and voluntarily took part in the study. The study protocol was approved by the Institutional Ethics Committee of Yangzhou University. No rewards or compensation were provided to the participants.

The inclusion criteria were as follows: (1) meeting the diagnostic criteria for stimulant use disorders according to the Diagnostic and Statistical Manual of Mental Disorders, Fifth Edition (DSM-5); (2) currently undergoing compulsory isolation and drug rehabilitation; (3) no history of brain injury or other organic lesions; (4) no history of mental illness, nor a family history of mental illness in immediate relatives; (5) normal intelligence; (6) normal color vision; (7) age below 50 years; and (8) primary school education or above.

### 2.2. Materials and Procedure

In the drug dot-probe task, the cues consisted of 20 drug-related and 20 neutral pictures, which were rated on a 1–9 scale by 20 women who did not participate in the subsequent formal experiment. The drug-related cues contained significantly more drug-related content (7.92 ± 2.33) than the neutral cues (1.54 ± 1.11, *p* < 0.001). In the emotion dot-probe task, the cues were selected from the Chinese Facial Affective Picture System [34] and included 20 negative and 20 neutral emotional face stimuli, with an equal number of male and female faces. There was no significant difference in the attractiveness ratings of the emotional stimuli (*p* = 0.76).

**Figure 1:**
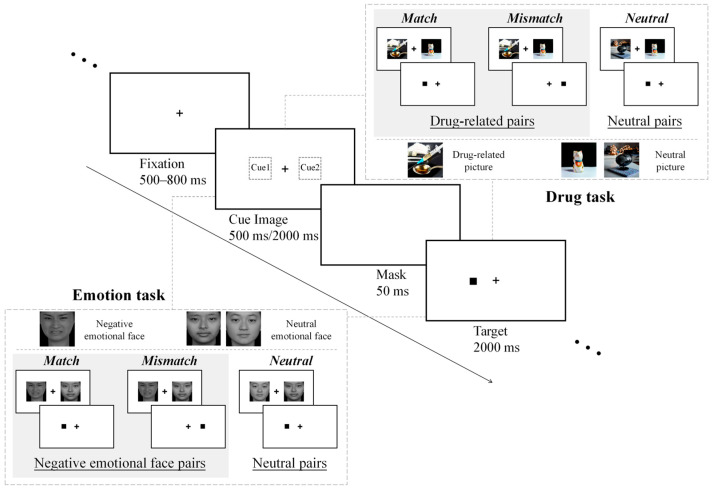
Task procedure. Each trial began with a central fixation cross (+) presented for 500–800 ms, followed by a cue display showing a pair of pictures for either 500 ms (short condition) or 2000 ms (long condition). After a 50 ms mask, a target stimulus (a solid black square) appeared on one side of the screen for up to 2000 ms and disappeared once the participant responded. The dashed boxes (top right: drug task; bottom left: emotion task) indicate the spatial relationship between cue images and the target: the match and mismatch conditions are part of the drug-related and negative emotional face pairs. Specifically, in the match condition, the drug-related picture or negative emotional face appeared on the same side as the target; in the mismatch condition, it appeared on the opposite side; and in the neutral condition, both cues were neutral pictures or neutral faces.

At the beginning of each trial, a fixation cross was presented at the center of the screen for 500–800 ms. Then, a pair of visual cues (220 × 220 pixels) was displayed on both sides of the screen. In the drug task, each pair could consist of one drug-related picture and one neutral picture, forming drug-related pairs, or two neutral pictures, forming neutral pairs. In the emotion task, each pair consisted of either one negative emotional face and one neutral face, forming negative emotional face pairs, or two neutral faces, forming neutral pairs. The cues were presented for either 500 ms (short cue condition) or 2000 ms (long cue condition). A mask was subsequently presented for 50 ms, followed by a target stimulus (a solid black square) that appeared for 2000 ms on either the left or right side of the screen, corresponding to the position of one of the visual cues. Participants were instructed to respond as quickly and accurately as possible to the target location by pressing “F” if it appeared on the left and “J” if it appeared on the right. See Figure 1.

According to the spatial relationship between the drug-related picture (in the drug task) or the negative emotional face (in the emotion task) and the subsequent target stimulus, three spatial conditions were defined: match, mismatch, and neutral. In the match condition, the drug-related or negative emotional face and the target stimulus appeared on the same side (left or right). In the mismatch condition, the drug-related or negative emotional face appeared on one side while the target stimulus appeared on the opposite side. In the neutral condition, both cues were neutral pictures or neutral faces. Cue images were presented under two duration conditions: short (500 ms) and long (2000 ms).

Thus, the experimental design comprised six conditions (3 spatial × 2 duration), with 10 trials per condition. The final experiment consisted of 10 practice trials with feedback and 120 experimental trials. Specifically, participants completed two separate blocks: a drug task block (60 trials) and an emotion task block (60 trials). The order of the two blocks was randomized across participants. Within each block, the trial sequence was randomized, and the presentation of drug-related pictures, negative emotional faces, and neutral pictures on the left or right side was counterbalanced. The total task duration was approximately 13 min.

The tasks were programmed and administered using E-Prime, with stimuli displayed on a 1280 × 960 pixel screen at a fixed viewing distance of 60 cm, yielding a visual angle of 5.4° × 7.3° for each picture.

### 2.3. Statistical Analysis

Reaction times (RTs) were recorded for each trial. Following established data-processing procedures [18,24], trials with incorrect responses, RTs shorter than 100 ms, or values exceeding ±2 SDs from each participant’s mean within a given condition were excluded. After these exclusions, an average of 94.25% of trials in both tasks were retained for analysis.

For each task, a 2 (cueing condition: short vs. long) × 3 (spatial condition: match, mismatch, neutral) repeated-measures ANOVA was conducted on RTs, with Obsessive Compulsive Drug Use Scale (OCDUS) scores included as a covariate. Partial *η*^2^ squared (*η*^2^_p_) was computed as the effect size for the *ANOVA*. When significant main effects or interactions were detected, multiple comparisons and simple effects analyses were performed with Bonferroni correction. Means and standard errors were reported for the primary outcomes (mean ± SE). We also examined the correlation between self-reported drug craving and performance on the task, specifically focusing on location-specific attentional bias [15]. This was assessed by calculating the difference in reaction times between the match and mismatch trials for each participant. Pearson’s correlation coefficient was used to determine the relationship between drug craving and attentional bias. All analyses were conducted using R (version 4.5), with a significance level set at 0.05.

## 3. Results

### 3.1. Drug Task

In the drug task, the main effect of spatial condition was significant, *F*(1.96, 98.24) = 4.05, *p* < 0.05, *η*^2^_p_ = 0.08. Specifically, RTs in the neutral condition (384.30 ± 8.87) were faster compared to the match condition (391.51 ± 9.76), *t* = 2.52, *p* < 0.05. The main effect of cue condition was significant, *F*(1, 50) = 151.66, *p* < 0.001, *η*^2^_p_ = 0.75. RTs in the 500 ms short cue condition (409.94 ± 9.19) were significantly slower than those in the 2000 ms long cue condition (368.22 ± 9.78), *t* = 12.32, *p* < 0.001.

**Figure 2:**
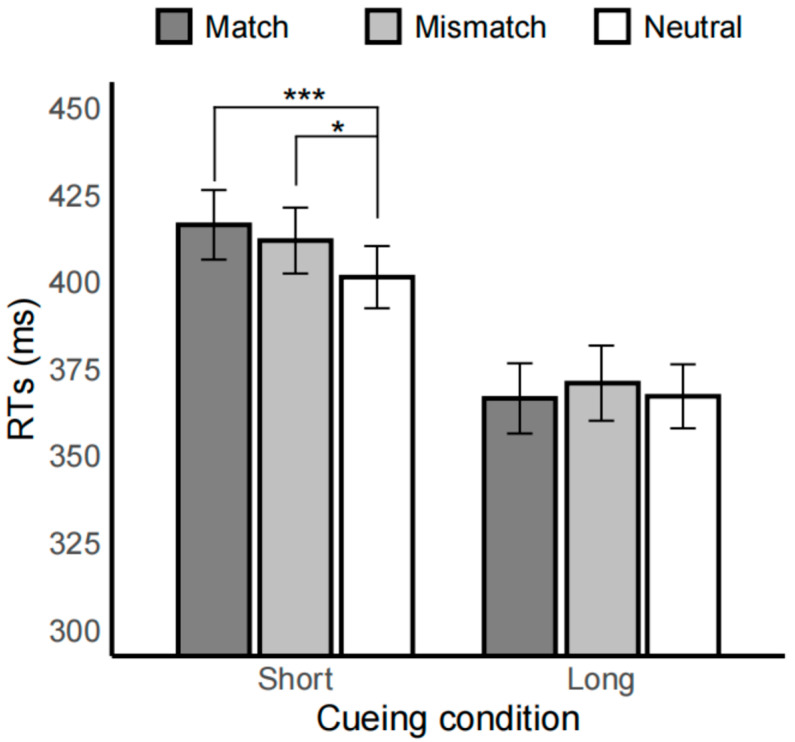
Bar plot of reaction times (RTs) under each condition in the drug task. Dark gray represents the match condition, light gray represents the mismatch condition, and white represents the neutral condition. The left side shows the short cue condition (500 ms), and the right side shows the long cue condition (2000 ms). Error bars represent standard errors; * indicates *p* < 0.05, and *** indicates *p* < 0.001.

Importantly, the interaction between spatial and cue condition was significant, *F*(1.78, 88.80) = 4.01, *p* < 0.05, *η*^2^_p_ = 0.07. Specifically, in the 500 ms short cue condition, RTs in the neutral condition (401.43 ± 8.94) were faster compared to both the mismatch (411.94 ± 9.47, *t* = 2.88, *p* < 0.05) and match conditions (416.46 ± 10.00, *t* = 4.27, *p* < 0.001). No significant difference was observed between the match and mismatch conditions (*t* = 0.76, *p* > 0.999). In the 2000 ms long cue condition, no significant differences were found between the spatial conditions (*ps* > 0.66), as shown in Figure 2.

**Figure 3:**
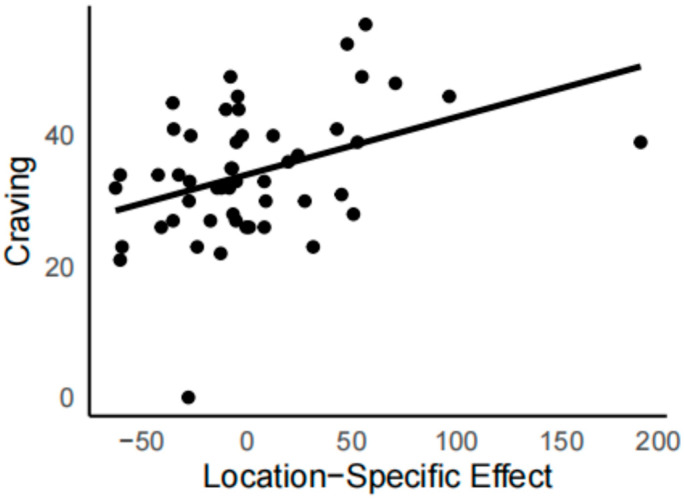
Scatter plot and correlation fit line of individuals’ drug location-specific attentional bias effect and craving at 500 ms. The location-specific effect was measured as the difference in reaction times between mismatch and match trials.

A correlation analysis was conducted to assess the relationship between both the global and location-specific effects and self-reported drug craving. The global effect was measured as the difference in reaction times between drug-related and neutral trials, while the location-specific effect was measured as the difference in reaction times between mismatch and match trials. Results showed that the location-specific effect was significantly positively correlated with drug craving in the 500 ms short cue condition (Figure 3), *r* = 0.40, *p* < 0.01, but not in the 2000 ms long cue condition, *r* = −0.09, *p* = 0.55. The global effect was not significantly correlated with drug craving in either the 500 ms (*r* = 0.10, *p* = 0.48) or 2000 ms cue condition (*r* = −0.01, *p* = 0.94).

### 3.2. Emotion Task

In the emotion task, the main effect of cue condition was significant, *F*(1, 50) = 129.41, *p* < 0.001, *η*^2^_p_ = 0.72. RTs in the 500 ms short cue condition (396.11 ± 8.68) were significantly slower than those in the 2000 ms long cue condition (356.63 ± 8.92), *t* = 11.38, *p* < 0.001. The main effect of spatial condition was not significant, *F*(1.97, 98.46) = 0.74, *p* = 0.48, *η*^2^_p_ = 0.02.

**Figure 4:**
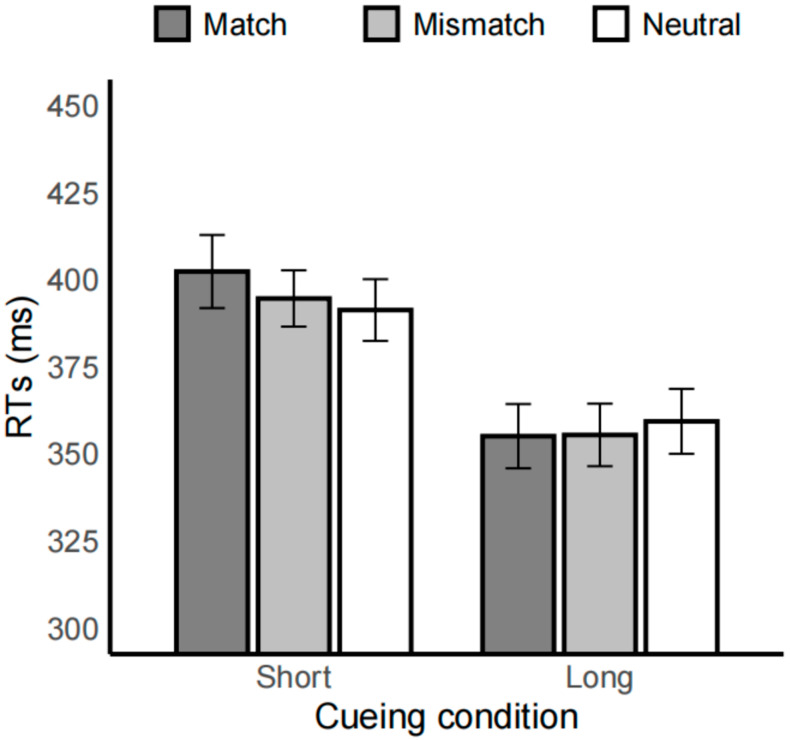
Bar plot of reaction times (RTs) under each condition in the emotion task. Dark gray represents the match condition, light gray represents the mismatch condition, and white represents the neutral condition. The left side shows the short cue condition (500 ms), and the right side shows the long cue condition (2000 ms). Error bars represent standard errors.

The interaction between spatial and cue condition was significant, *F*(1.96, 97.82) = 3.27, *p* < 0.05, *η*^2^_p_ = 0.06. Specifically, in the match condition (402.38 ± 10.45 vs. 355.10 ± 9.23), mismatch condition (394.64 ± 8.09 vs. 355.45 ± 8.98), and neutral condition (391.32 ± 8.86 vs. 359.35 ± 9.33), RTs in the 500 ms short cue condition were significantly slower than those in the 2000 ms long cue condition (*ps* < 0.001). In both the 500 ms short cue and 2000 ms long cue conditions, no significant differences were found between the match, mismatch, and neutral conditions (*ps* > 0.16), as shown in Figure 4.

The global effect was measured as the difference in reaction times between negative emotional face pairs and neutral trials. The location-specific effect showed no significant correlation with drug craving in either the 500 ms (*r* = −0.09, *p* = 0.54) or 2000 ms cue condition (*r* = −0.20, *p* = 0.17). Similarly, the global effect was not significantly correlated with drug craving in the 500 ms (*r* = 0.03, *p* = 0.83) or 2000 ms cue condition (*r* = −0.04, *p* = 0.78).

## 4. Discussion

The present study aimed to investigate the effects of cue duration and spatial congruency on attentional bias toward drug-related and negative emotional stimuli in female individuals with drug dependence. Consistent with our hypotheses, global drug-related attentional bias emerged during short cue exposure (500 ms) rather than during the sustained attention stage (2000 ms). Specifically, reaction times to the target were slowed whenever the cue contained drug-related stimuli, regardless of its spatial location. This attentional bias was specific to drug-related cues and did not extend to negative emotional stimuli.

During short cue exposure (500 ms), participants showed interference from drug-related pairs in the drug-related dot-probe task, with reaction times slowed regardless of whether the drug cue’s location matched the probe. This result is consistent with previous research, suggesting that a global attentional bias exists in female individuals with drug dependence [15]. Specifically, slower reaction times were observed when drug-related cues and targets appeared in different locations, which is partly in line with findings from male populations under similar conditions. A recent study in individuals with methamphetamine-use disorder similarly reported significantly slower reaction times when drug cues appeared on one side and the target on the opposite side, compared with trials in which cues on both sides were neutral [18]. Additionally, the present study found that reaction times were also slowed when drug-related cues and targets were spatially matched, consistent with baseline data reported by Alireza Shahbabaie et al. [25]. In contrast, during the sustained attention stage (2000 ms), drug-related cues did not elicit any form of attentional bias, consistent with previous findings. One study using a dot-probe task with a 2000 ms cue exposure similarly reported no attentional bias toward drug-related cues [24]. However, unlike the findings in male populations, He et al. did not observe slower reaction times when drug-related cues were spatially matched with the probe, a result observed in the current female sample [18]. Although a location-specific attentional bias (the difference between match and mismatch conditions) was not observed at the group level in this study, it exhibited a positive correlation with self-reported drug craving under the 500 ms task condition. From an individual perspective (Figure 3), some participants showed little to no location-specific effect and even demonstrated effects in the opposite direction, which corresponded to their low levels of craving. Higher drug craving was associated with greater location-specific attentional bias, whereas no such relationship was observed under the 2000 ms cue condition. This also suggests that the relationship between attentional bias and craving may be limited and context-dependent [7,8,9]. These findings collectively indicate that the influence of drug-related cues on female attentional processes is temporally constrained, primarily affecting the early orienting stage rather than sustained attention. When drug-related stimuli appear, reaction times to the probe are slowed.

The observed attentional bias toward short-duration drug cues once again confirms both the incentive sensitization model of addiction and load theory [20,22,23]. Individuals with drug dependence, due to prolonged exposure, are involuntarily captured by drug-related stimuli [35]. Drugs preferentially capture attention as reward-predictive cues, even when the associated reward is no longer present, reflecting a form of irrational attentional bias [36]. When drug and neutral cues were presented simultaneously, drug cues preferentially captured the automatic attention of individuals with substance dependence, even when the reward was not immediately obtainable, thereby consuming a portion of attentional resources [19]. The higher level of interference caused by drug-related cues in individuals with addiction activates drug-related thoughts and concerns that need to be suppressed, leading to greater allocation of attentional resources, which subsequently slows reaction times to target locations [16]. At longer cue exposures of 2000 ms, individuals with substance dependence accumulated greater perceptual load. However, because the drug cues were task-irrelevant under these conditions, the increased load was largely filtered out, preventing interference with subsequent task performance [22,23]. This interpretation is further supported by the overall faster reaction times observed in the 2000 ms condition compared with the 500 ms condition, consistent with previous findings. Prior research likewise reported shorter reaction times under 2000 ms cue exposure relative to 500 ms (415 vs. 437 ms), including in neutral picture trials (407 vs. 435 ms) [24]. Studies on the relationship between foreperiod length and reaction speed have also shown that, within a certain range (typically tens of milliseconds to a few seconds), longer preparation times are associated with faster responses [37,38,39], suggesting that prolonged exposure provides sufficient time for filtering and adaptation to task demands.

No reaction time differences were observed for negatively valenced emotional cues, indicating that the attentional bias in individuals with substance dependence did not extend to these stimuli. In other words, the attentional bias observed in this population appears to be specific to drug-related stimuli rather than reflecting a generalized hypervigilance to emotionally salient information. This finding is consistent with previous research: behaviorally, some studies reported no reaction time differences [18], and electrophysiologically, early posterior negativity (EPN) amplitudes did not differ significantly between emotional and neutral stimuli in substance-dependent individuals [28]. Taken together, these results underscore the stimulus-specific nature of attentional bias in substance dependence. They highlight that drug-related cues, rather than general emotional cues, preferentially engage attentional resources. This engagement, in turn, influences task performance.

The present study makes several contributions to the literature on attentional bias in drug dependence. First, by including both 500 ms and 2000 ms cue exposure durations, as well as both spatially matched and mismatched cue-target arrangements, this study provides a more systematic understanding of the temporal and spatial dynamics of attentional bias [18,24]. Our results demonstrate that global drug-related attentional bias is most pronounced during the short cue exposure attention, supporting the incentive sensitization model and extending prior findings. Attentional capture by drug cues occurred regardless of spatial congruency during short cue exposures [25], suggesting that the perception and processing of drug-related information may be a key factor driving attentional bias. Second, this study specifically examined female individuals with drug dependence, contributing to the limited research on attentional bias in this population. Most prior research has been conducted with predominantly male samples [21,24], limiting generalizability. Female drug-dependent individuals also exhibited attentional bias toward drug-related cues, consistent with the overall patterns previously reported in male samples. Finally, by including both drug-related and negative emotional cues, the present study clarifies the specificity of attentional bias in drug dependence. Research on the relationship between affordances, attention, and emotions is crucial in understanding how environmental factors shape cognitive and emotional responses [18,40,41]. Finally, these findings have important clinical implications for designing interventions like attentional bias modification (ABM) to target automatic attentional capture by drug-related cues [42]. Tailoring these interventions to female populations could improve treatment outcomes, addressing the specific challenges they face.

Despite these contributions, several limitations should be noted and addressed in future research. First, only two cue exposure durations (500 ms and 2000 ms) were examined in the present study. We acknowledge that the relatively small number of trials per condition (10) may compromise the reliability of the response time measures. Future studies should aim to include a larger number of trials to ensure more robust data. While these durations provided valuable insights, additional intermediate durations (e.g., 1000–1500 ms) would allow for a more precise characterization of the temporal dynamics of attentional bias. Future studies could explore a wider range of cue exposure times to better understand the nuanced stages of attentional processing [21]. Second, the current evidence is purely behavioral, which limits our ability to infer the underlying cognitive and neural mechanisms. While we interpret the effects in terms of incentive sensitization and load theory, reliance on reaction times alone makes the interpretation speculative. Future research should incorporate electrophysiological methods, such as ERP or eye-tracking, to provide more evidence of the neural processes involved in attentional capture and resource allocation. Third, considering the sex differences in dopamine release in the brain in response to drug consumption, as well as the effects of the menstrual cycle on the subjective effects of the drug, these factors were not addressed in the present study. Future research should incorporate more physiological indicators and systematically account for factors such as the menstrual cycle in order to further explore attentional bias in female populations. Additionally, the absence of a healthy control group of drug-free females makes it difficult to determine whether the observed attentional bias is specific to individuals with drug dependence or reflects a broader pattern. Including a control group would strengthen the interpretation of the findings and provide a more comprehensive understanding of the attentional processes at play. Finally, we recommend that future research include more diverse samples, including participants from various treatment centers and cultural backgrounds, to better understand how generalizable these findings are across different contexts and populations.

## 5. Conclusions

In conclusion, the present study demonstrates that female individuals with drug dependence exhibit a drug-specific global attentional bias that is most pronounced during the short cue exposure attention, as reflected by slowed reaction times in response to drug-related cues under short-duration exposure. This bias occurs regardless of spatial congruency and does not extend to negative emotional stimuli, indicating the stimulus-specific nature of attentional processing in this population. These results enhance our understanding of the temporal and spatial dynamics of attentional bias in drug dependence. They may also inform the development of targeted interventions. Such interventions could help reduce attentional bias and improve cognitive and behavioral outcomes in this population.

## Figures and Tables

**Table 1:** Demographic characteristics and drug use measures (*n* = 51).

Measure	Mean (Standard Deviation)
Education level (junior high school or below/senior high school)	42/9
Marital status (married/unmarried/cohabiting/divorced/separated)	6/30/4/10/1
Age	24.71 (7.58)
Age at first drug use	17.94 (3.18)
Mean duration of drug use	5.51 (4.82)
OCDUS	34.94 (8.65)

Abbreviation: OCDUS, Obsessive Compulsive Drug Use Scale.

## Data Availability

The original contributions presented in the study are included in the article; further inquiries can be directed to the corresponding authors. Due to privacy and ethical restrictions, the data are not publicly available.

## Abbreviation

The following abbreviation is used in this manuscript:

OCDUS	Obsessive Compulsive Drug Use Scale
